# Static Electricity-Induced Luminescence Materials for Charge Sensing

**DOI:** 10.3390/ma19132709

**Published:** 2026-06-24

**Authors:** Tomoya Sato, Taiga Eguchi, Nanami Ishizu, Yuki Fujio, Kazuya Kikunaga

**Affiliations:** 1 Sensing Technology Research Institute, National Institute of Advanced Industrial Science and Technology, 807-1 Shuku-Machi, Tosu 841-0052, Japan; eguchi-0616@aist.go.jp (T.E.); yuki-fujio@aist.go.jp (Y.F.); k-kikunaga@aist.go.jp (K.K.); 2 Faculty of Science and Engineering, Saga University, 1 Honjo, Saga 840-8502, Japan

**Keywords:** static electricity, electric charge distribution, static electricity-induced luminescence, SrAl_2_O_4_:Eu^2+^

## Abstract

Static electricity-induced luminescence (SEL) materials exhibit luminescence in response to minute electrical charges and therefore have potential for application in self-powered charge-detection sensors that operate without an external power source. However, important aspects of their luminescence mechanism and the associated material properties remain insufficiently understood. In this study, SEL films based on SrAl_2_O_4_:Eu^2+^ were evaluated, and the effects of SrAl_2_O_4_:Eu^2+^ concentration and applied voltage on the luminescence behavior were quantitatively investigated. The results showed that the SEL intensity increased in proportion to the square of the applied voltage, while the SEL luminescence area increased monotonically with increasing voltage. These results suggest that the SEL intensity and SEL area may reflect the amount of discharge–charge from the needle electrode and the charge distribution on the film surface, respectively. In addition, increasing the SEL phosphor content enhanced the luminescence intensity, whereas no significant effect was observed on the relative change in luminescence area with applied voltage. Collectively, these findings provide fundamental insights for the design of charge-detection sensors based on SrAl_2_O_4_:Eu^2+^.

## 1. Introduction

In industrial environments, static electricity may lead to reduced product productivity and reliability, as well as to accidents, and therefore electrostatic control is considered an important issue. Static electricity arises from an imbalance of electric charge generated by friction, contact, or separation between objects. For example, when a positively charged insulator (e.g., the human body) approaches a grounded conductor, the strong electric field between them can trigger dielectric breakdown and a transient current. This event is known as electrostatic discharge (ESD) and, depending on its magnitude, may be accompanied by audible or visible effects. Electrostatic phenomena not only damage electronic devices and cause equipment malfunctions through ESD, but also promote the adhesion of foreign particles via electrostatic attraction (ESA) [1,2]. Furthermore, they can initiate catastrophic events, including ignition of flammable materials and dust-induced explosions and fires [3,4]. Accordingly, to sustain productivity and ensure safety in manufacturing environments, highly effective electrostatic control measures are essential.

However, static electricity is inherently invisible, and its occurrence is difficult to detect or predict instantaneously and accurately. Effective countermeasures, therefore, require precise assessment of the charge state. Conventional field-mill [5] and electrostatic induction-type [6,7] electric field meters enable convenient evaluation, but they typically report area-averaged values and thus cannot capture two-dimensional charge distributions in a single measurement. To address this limitation, two-dimensional mapping has been pursued using scanning probe microscopy (SPM) [2,8,9] and scanning systems based on line-array sensors [10,11]. In addition, imaging approaches that integrate electro-optical effects (e.g., the Pockels and Kerr effects) with imaging devices have been proposed [12,13,14]. Despite these efforts, existing techniques still face limitations in spatial resolution and detection sensitivity, and real-time operation remains severely constrained. Consequently, there is a strong need for new measurement technologies that can visualize otherwise invisible static electricity and enable rapid, instantaneous detection and evaluation.

Europium(II)-doped strontium aluminate (SrAl_2_O_4_:Eu^2+^) exhibits excellent phosphorescence and mechanoluminescence and has been extensively studied for use in light-emitting devices [15,16,17,18]. SrAl_2_O_4_:Eu^2+^ shows a broad emission spectrum centered around 520 nm, primarily originating from Eu^2+^ [18,19,20]. Kikunaga et al. recently reported that faint electrostatic discharge generated when a charged finger approaches SrAl_2_O_4_:Eu^2+^ can be observed as luminescence [21]. This luminescence phenomenon, detectable even for low-energy electrostatic charges, was termed “static electricity-induced luminescence” (SEL). Using SrAl_2_O_4_:Eu^2+^ as an SEL material, static electricity has been visualized under atmospheric pressure at room temperature [21]. Although the detailed mechanism of SEL has not yet been fully elucidated, it has been discussed based on a process similar to that of mechanoluminescence [16,19,21,22,23]. In SrAl_2_O_4_:Eu^2+^, electrons of Eu^2+^ are excited by the absorption of ultraviolet to blue light. A portion of the excited electrons rapidly relaxes to the ground state of Eu^2+^, emitting green light in the process, whereas the remaining excited electrons are trapped at defect states. Subsequently, external stimuli such as electrons or ions generated during charging and discharging release electrons trapped at shallow defect states, and these released electrons migrate to Eu^2+^ and transition to the ground state, resulting in the luminescence observed as SEL. Furthermore, as Eu^2+^ returns to the ground state via SEL, the luminescence ceases. However, SEL can be observed repeatedly when Eu^2+^ can be re-excited through the absorption of ultraviolet to blue light.

A related charge-excitation phenomenon is cathodoluminescence (CL), which has long been used in materials analysis, such as the detection of trace impurities and lattice defects, using electron microscopes [24,25]. However, CL requires direct irradiation of a phosphor with high-energy electron beams under vacuum, which limits its applicability for visualizing static electricity that commonly occurs in the atmosphere. Conversely, SEL enables detection of static electricity in real-world environments because SrAl_2_O_4_:Eu^2+^ can emit high-intensity light in response to low-energy charges. Importantly, SEL does not require large-scale instrumentation or an external power source to induce luminescence. Consequently, SEL is expected to enable the development of self-contained charge detection sensors that are not achievable with conventional approaches.

Previous studies documented luminescence immediately after corona discharge was induced using an electrostatic gun directed at an SEL film; the luminescent region also expanded over time [21,26]. However, studies on SEL films are still limited, and the luminescence behavior has so far been discussed only qualitatively. In addition, because SrAl_2_O_4_:Eu^2+^ is a ceramic particle, SEL films are generally fabricated by mixing it with a binder resin [21,27]. Although the SrAl_2_O_4_:Eu^2+^ content is expected to strongly affect the luminescence properties, this effect has not yet been systematically evaluated. Therefore, to apply charge-responsive SEL films to charge-sensing applications, it is important to quantitatively clarify the relationships among luminescence indicators (e.g., SEL intensity and luminescent area), discharge conditions, and material composition. In this study, the luminescence characteristics of SEL films based on SrAl_2_O_4_:Eu^2+^ were systematically and quantitatively evaluated under a wide range of applied high voltages between the SEL film and a needle electrode by means of image analysis. Furthermore, multiple SEL films with different SrAl_2_O_4_:Eu^2+^ contents were prepared to investigate the influence of composition on luminescence characteristics and their interrelationships.

## 2. Materials and Methods

### 2.1. Sample Preparation

Commercial SrAl_2_O_4_:Eu^2+^ powder (ML032, Sakai Chemical Industry Co., Ltd., Sakai City, Japan) was used as the SEL material. The particle size of the SrAl_2_O_4_:Eu^2+^ powder was controlled by the manufacturer to be several μm. Because SrAl_2_O_4_:Eu^2+^ consists of ceramic particles, directly forming a uniform film from the powder on a substrate is challenging. To enable immobilization on the substrate, the particles were mixed with epoxy resin (PS ink series, Teikoku Printing Inks Mfg. Co., Ltd., Tokyo, Japan), which served as a binder. SEL films were prepared by screen-printing a mixture of SrAl_2_O_4_:Eu^2+^ adjusted to a weight fraction of 10–70% relative to the epoxy resin onto 12-μm-thick aluminum foil (Professional-use aluminum foil, UACJ Foil Corp., Tokyo, Japan). Subsequently, the films were subjected to a drying process in a constant-temperature chamber maintained at 130 °C for 1 h. It is imperative to consider the impact of residual solvents present within the SEL film, as their presence can potentially compromise the luminescent properties. In order to mitigate this potential issue, the curing temperature and time for the film were set to 130 °C with the objective of ensuring the complete curing of the epoxy resin, while also facilitating the complete evaporation of any residual solvents.

All SEL films utilized in this study were 100 × 100 mm^2^. The thickness of each film was measured using an eddy current film thickness gauge (LH-373, Kette Electric Laboratory Co., Ltd., Tokyo, Japan). Film thickness was measured at multiple locations within each SEL film, and no significant variation in thickness was observed; the average film thickness was approximately 70 μm. Based on these results, it is considered that the SEL films utilized in this study generally exhibit uniformity.

### 2.2. Experimental Setup

**Figure 1:**
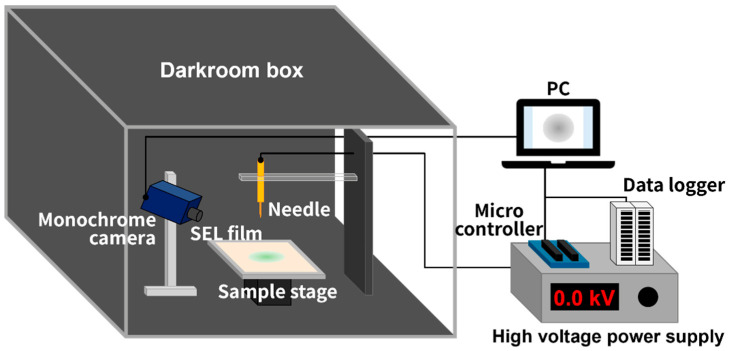
Overview of the SEL characteristics evaluation system employed in this study.

An overview of the luminescence evaluation system is shown in Figure 1. Each SEL film, which had been formed on aluminum foil, was affixed to a grounded sample stage. A needle electrode (1 mm in diameter) connected to a high-voltage power supply (MODEL 610E, Trek, Waterloo, WI, USA) was positioned above the SEL film, and luminescence under applied voltage was recorded with a monochrome camera (U3-3860SE-M-GL, IDS, Obersulm, Germany). The needle-to-film distance was fixed at 45 mm. Pulse voltages of 4–10 kV (pulse width, 200 ms) were applied to the electrode under microcontroller control (Arduino-uno, Arduino, Monza, Italy). The application of these voltages was recorded by a data logger (NR-500, KEYENCE Corp., Osaka, Japan). In order to ensure stable and reproducible discharge behavior, the needle-to-film distance and the film and the applied voltage were selected based on our previous study [26].

The image size, frame rate, and bit depth of the monochrome camera were configured to 550 × 320 pixels, 60 fps, and 12-bit, respectively. The gain value was determined through a process of trial and error, ultimately yielding a value of 3.54. Image acquisition was conducted using IDS peak Cockpit 1.7.0.0 (IDS Imaging Development Systems GmbH, Obersulm, Germany). Within the software framework, the 12-bit images were automatically expanded to 16-bit depth and stored. Luminescence characteristics, including intensity we and luminescence area, were evaluated at each applied voltage. The acquired images were analyzed using in-house software developed with the open-source library OpenCV 4.6.0 [28] to quantify these luminescence characteristics.

### 2.3. Image Analysis Procedure

In this study, image analysis was performed on the acquired images to systematically compare the luminescence characteristics of the SEL films. Initially, 300 frames corresponding to the 5 s immediately preceding the onset of luminescence were extracted from the acquired time-series images. The baseline was determined by evaluating the luminance distribution of these frames. To ensure inter-experimental reproducibility and consistency in baseline levels, differential correction was applied to the maximum luminance value obtained in each experiment. Subsequently, the baseline-corrected image sequence was time-integrated at intervals of approximately 16.67 ms to generate an integrated image. In this study, a unified reference range for the luminance distribution was established using the integrated images obtained at the minimum (4 kV) and maximum (10 kV) applied voltages, and min–max normalization was then applied to each integrated image acquired under different measurement conditions.

This normalization method may reduce the visual prominence of weak luminescence observed under low-voltage conditions. However, the relative intensity relationships within and between datasets are preserved. Furthermore, the utilization of time-integrated images improves the signal-to-noise ratio, thereby facilitating the quantitative detection of low-intensity luminescence. Therefore, this normalization procedure does not compromise the validity of comparative analysis; rather, it enables a consistent and physically meaningful evaluation of luminescence characteristics under different applied-voltage conditions.

## 3. Results and Discussion

### 3.1. Luminescence Properties of SEL Films

**Figure 2:**
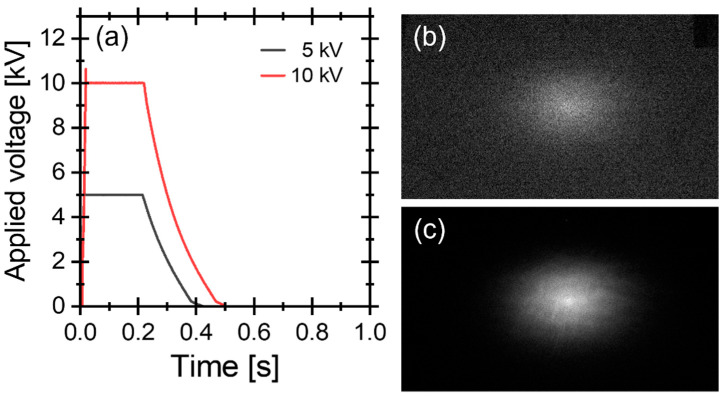
(**a**) Pulse voltage waveforms of 5 and 10 kV. SEL images when (**b**) 5 kV and (**c**) 10 kV were applied to the needle electrode.

The luminescence properties of the SEL films are influenced by two factors: the magnitude of the voltage applied to the needle electrode and the concentration of Sr SrAl_2_O_4_:Eu^2+^. First, a thorough investigation was conducted into the voltage dependence of the luminescence properties. For this study, a 50% SrAl_2_O_4_:Eu^2+^ film was selected as the reference sample because it was prepared by mixing the binder resin and SrAl_2_O_4_:Eu^2+^ in a 1:1 weight ratio, which represents the simplest composition ratio. To evaluate the luminescence characteristics, pulse voltages of 5 and 10 kV, as shown in Figure 2a, were applied via a needle electrode to an SEL film containing 50% SrAl_2_O_4_:Eu^2+^. The corresponding SEL images acquired under applied voltage are shown in Figure 2b,c, respectively. The images of Figure 2b,c were normalized to the maximum brightness observed at 5 kV and 10 kV, respectively. As shown in Figure 2b,c, SEL emission from the film was observed upon voltage application to the needle electrode. This SEL is considered to originate from the release of electrons trapped at shallow defect states by stimulation from low-energy charges emitted from the needle tip during pulse-voltage application, followed by their migration to Eu^2+^ and recombination with holes. This observation is consistent with previous reports [21,26,27]. Moreover, similar luminescence behavior of SEL films was also observed in preliminary experiments, indicating that this luminescence phenomenon exhibits good reproducibility.

A quantitative comparison of the luminescence intensity and luminescence area of both samples was performed by image analysis. The images shown in Figure 2b,c were acquired using a monochrome camera positioned at an angle of approximately 50° from the normal direction of the sample surface. Therefore, a projection transformation was applied to all acquired images to correct them into bird’s-eye views taken directly from above the sample. Integrated luminescence images were then generated for each measurement and normalized to the maximum SEL intensity at 10 kV to place all images on a common intensity scale.

**Figure 3:**
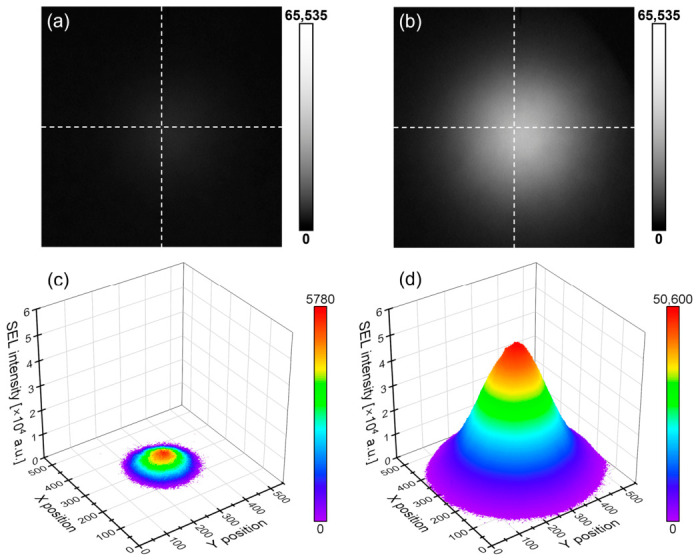
Integrated luminescence image captured when (**a**) 5 kV and (**b**) 10 kV pulse voltages were applied to the needle electrode in a 50% SrAl_2_O_4_:Eu^2+^ sample. 3D plots of SEL intensity distribution when (**c**) 5 kV and (**d**) 10 kV pulse voltages were applied to the needle electrode in a sample with 50% SrAl_2_O_4_:Eu^2+^.

As shown in Figure 3a,b, integrated luminescence images were obtained under applied voltages of 5 and 10 kV, respectively. The center of each image corresponds to the position directly beneath the needle electrode. At 10 kV (Figure 3b), luminescence clearly spreads outward from the region beneath the electrode. Conversely, at 5 kV (Figure 3a), luminescence is not readily visible in the two-dimensional image. This occurs because the images were normalized to the maximum SEL intensity at 10 kV; consequently, the relatively weaker luminescence at 5 kV was assigned to lower tonal values, resulting in a tonal range that is no longer discernible to the human eye. Consistent with this interpretation, the 3D intensity distribution at 5 kV (Figure 3c) shows that luminescence is concentrated primarily beneath the needle electrode, similar to the 10 kV case (Figure 3d). The peak SEL intensity directly beneath the electrode is attributed to the locally maximized electric field in this region. In this configuration, the application of a high voltage to the needle electrode induces charge emission from the tip, and the resulting charges are transported toward the luminescent film according to the electric-field distribution. Because the electric field is strongest where the electrode–film separation is smallest (i.e., directly beneath the tip), most charges are expected to be focused toward the center, producing the maximum SEL intensity in that region.

**Figure 4:**
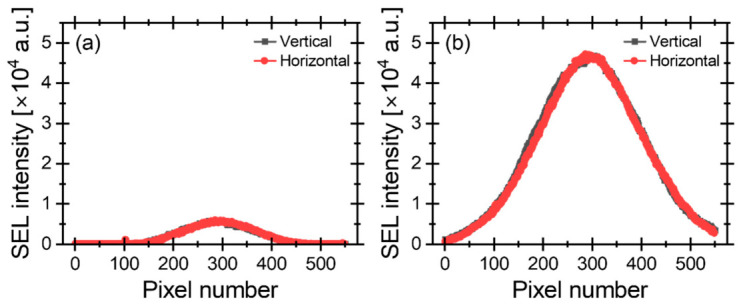
Comparison of vertical and horizontal SEL intensity profiles through the center of the integrated luminescence image (white dashed lines in Figure 3a,b). Panels (**a**,**b**) show the SEL intensity profiles obtained when (**a**) 5 kV and (**b**) 10 kV are applied to the needle electrode, respectively.

Subsequently, an examination was conducted to ascertain SEL spatial distribution. The integrated images in Figure 3a,b were used to extract pixel-by-pixel SEL intensity profiles along vertical and horizontal lines passing through the image center. These profiles are presented in Figure 4. As shown in Figure 4, the vertical and horizontal intensity distributions agree closely for both 5 and 10 kV, indicating radial symmetry. The same trend was also observed under additional applied-voltage conditions using an SEL film with 50% SrAl_2_O_4_:Eu^2+^ (Figure S1). These results indicate that SEL induced by voltage application spreads isotropically from a luminescence center located directly beneath the needle electrode, with intensity decreasing gradually with increasing distance from the center. Because SEL originates from SrAl_2_O_4_:Eu^2+^ in response to incident charge, the results in Figure 3 and Figure 4 suggest that the amount of charge contributing to SEL decreases with increasing distance from the luminescence center.

The dependence of luminescence intensity and luminescence characteristics on applied voltage in SEL films containing 50% SrAl_2_O_4_:Eu^2+^ was also investigated. In a needle–plate electrode system, corona discharge occurs at low voltages because the electric field is concentrated at the needle tip. As is well established, the discharge current (I) is positively correlated with the applied voltage (V) in needle–plate systems. This relationship can be described by the Townsend equation [29,30]

(1) $$I=CV\left(V-V_{0}\right),$$

where V_0_ represents the breakdown voltage, and C is a constant influenced by various factors, including the needle-tip radius of curvature, gas type, humidity, and charge mobility. Under specific conditions (e.g., a fixed gap length), an alternative model has also been proposed, as shown below [31,32]

(2) $$I=C{\left(V-V_{0}\right)}^{m},$$

where m is a constant ranging from 1.5 to 2.0. Although many empirical and semi-empirical expressions have been reported, the discharge current generally tends to scale with the square of the applied voltage. Furthermore, because the total charge transferred during corona discharge corresponds to the time integral of the current, these relationships imply that the discharged charge also scales approximately with the square of the applied voltage.

**Figure 5:**
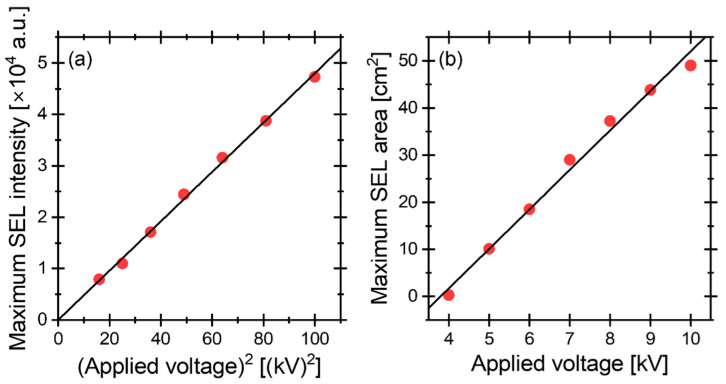
(**a**) Maximum SEL intensity of the SEL 50% film as a function of the square of the applied voltage. (**b**) Luminescence area of the SEL 50% film as a function of the applied voltage.

Given that SEL is triggered by charge injection into the film, the maximum SEL intensity was plotted as a function of the square of the applied voltage, and the relationship between the applied voltage and SEL intensity was examined. The results are shown in Figure 5a. As shown in Figure 5a, the SEL intensity exhibited a tendency to increase in proportion to the square of the applied voltage. Furthermore, the vertical and horizontal SEL intensity profiles obtained at applied voltages of 4, 6, 7, 8, and 9 kV were compared to investigate the voltage dependence of the luminescence area. Consequently, under all conditions, a radially distributed luminescence pattern centered directly beneath the needle electrode was observed (Figure S1). Accordingly, assuming that the luminescence region is a perfect circle, the luminescence area at each applied voltage was calculated based on the diameter estimated from the widths of the rising portions of the vertical and horizontal intensity distributions. The results are shown in Figure 5b. As is clear from Figure 5b, the luminescence area increases approximately in proportion to the voltage applied to the needle electrode.

Here, the voltage dependences of the SEL intensity and SEL area are discussed. In general, when a high voltage is applied to a needle electrode, a localized strong electric field is formed at the electrode tip, and charges are emitted as the discharge progresses. These charges then migrate toward the opposing plate electrode along the formed electric field. However, during this process, the electric field lines are pushed outward from the center toward the periphery due to the space charge effect [33]. Consequently, the range of charges reaching the plate increases, reaching a maximum at the center and rapidly decreasing toward the periphery. Theoretical studies have suggested that this effect gives rise to a bell-shaped current density distribution [34,35], and calculations based on this framework have been reported to show good overall agreement with experimental results [34,36]. In light of these previous studies, these results suggest that the changes in SEL intensity and SEL area observed with increasing applied voltage to the needle electrode may represent responses to changes in the amount of discharge–charge and the charge distribution on the SEL film surface, respectively.

Conventionally, discharge phenomena have been evaluated by detecting weak optical emission generated by molecular ionization and excitation caused by electron collisions near a needle electrode [37,38,39]. However, because such emission occurs mainly in the ultraviolet region, direct observation with the naked eye is difficult. Moreover, weak discharges that do not produce distinct optical emission, such as those occurring in the dark-discharge region, are difficult to detect even when electrons and ions are generated. In addition, Nie et al. proposed a high-voltage sensing approach based on the electroluminescence of ZnS:Cu, in which localized luminescence is generated by applying an external high voltage to an EL coating [40]. However, because this luminescence relies on an externally applied high electric field, the method has limitations in detecting low-electric-field regions or minute charged states. In contrast, SEL emits visible light in response to discharge-induced charges and therefore offers the advantage of directly and simply visualizing charge-related phenomena without requiring a dedicated detection device or an external power source for luminescence excitation. The results obtained in this study further suggest that, when combined with image analysis, SEL can provide simultaneous information on both the amount of discharge–charge and its spatial distribution on the film surface. Furthermore, SEL may be useful for detecting the initial stages of discharge processes corresponding to the dark-discharge region [21]. Thus, the SEL-based approach has the potential to develop into a simple charge-detection and visualization technology for minute discharges or electrification events at arbitrary locations, which are difficult to capture using conventional techniques. Although further studies are required to quantitatively evaluate detection sensitivity, spatial resolution, and response characteristics and to establish design guidelines for charge-detection sensors, the present results indicate that SEL materials are promising candidates for realizing direct charge-detection technologies.

### 3.2. Correlation Between the SrAl_2_O_4_:Eu^2+^ Content and Luminescence Properties

**Figure 6:**
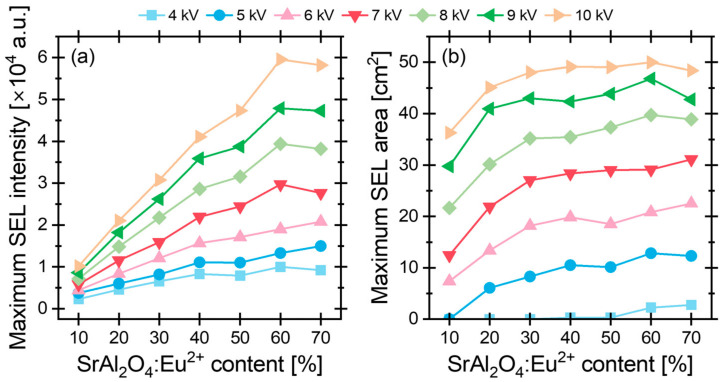
Changes in (**a**) maximum SEL intensity and (**b**) maximum SEL area as a function of SrAl_2_O_4_:Eu^2+^ content.

For the application of SEL materials to charge-detection sensors, the SrAl_2_O_4_:Eu^2+^ concentration in the film is likely to be a key parameter for optimizing sensor performance, such as signal intensity and charge-detection sensitivity. To examine this dependence, SEL films with varying SrAl_2_O_4_:Eu^2+^ ratios were fabricated and examined to determine the correlation between these ratios and the luminescence characteristics of the films. As shown in Figure 6, the maximum SEL intensity and luminescence area were plotted against the SrAl_2_O_4_:Eu^2+^ content in each film.

As shown in Figure 6a, at a fixed applied voltage, the maximum SEL intensity increased approximately proportionally with increasing SrAl_2_O_4_:Eu^2+^ content. Moreover, the rate of increase grows with applied voltage. These findings suggest that increasing the content ratio may enhance the charge-induced luminescence response. At high applied voltages, the steeper dependence of SEL intensity on phosphor content is considered to arise from the relationship between the applied voltage and the discharge–charge supplied to the SEL film. As mentioned earlier, discharge current has generally been reported to exhibit a tendency to be proportional to the square of the applied voltage [29,30,31,32]; therefore, the discharge–charge is also expected to show a similar voltage dependence. Because the amount of charge reaching the SEL film increases quadratically with increasing applied voltage, a larger number of SrAl_2_O_4_:Eu^2+^ particles may respond, resulting in a steeper dependence of SEL intensity on phosphor content under high voltage conditions than under low voltage conditions.

In contrast, when the SrAl_2_O_4_:Eu^2+^ content was 60% and 70%, the SEL intensity tended to remain nearly constant. One possible explanation for this behavior is that, under the present experimental conditions, SrAl_2_O_4_:Eu^2+^ was present in excess relative to the amount of charge injected into the film. The SEL observed in this study is considered to be generated by an external stimulus associated with charge injection into the film during discharge, whereby charges trapped at shallow defect states are released and subsequently migrate to the luminescent center, Eu^2+^, resulting in light emission. In other words, these results suggest that, within the experimental range investigated in this study, when the phosphor content exceeds 60%, the number of Eu^2+^ luminescent centers becomes relatively excessive with respect to the amount of charge injected into the film.

Conversely, the luminescent area showed a different dependence than SEL intensity. At 4 kV, luminescence was detected at low SrAl_2_O_4_:Eu^2+^ contents; however, it was not possible to quantitatively evaluate its spatial extent. At 5 kV and above, luminescence induced by the corona discharge at the needle-electrode tip was observed for all doping concentrations, enabling quantitative evaluation of the luminescent area. The luminescent area increased with SrAl_2_O_4_:Eu^2+^ content up to 30%, but tended to plateau once the content exceeded 30%. As noted above, the luminescent area is hypothesized to reflect the spatial charge distribution on the SEL film. At a fixed needle-electrode voltage, the charge distribution on the film surface is expected to remain largely unchanged, despite differences in film composition. Collectively, these findings indicate that, for SEL films containing ≥ 30% SrAl_2_O_4_:Eu^2+^, sufficient SrAl_2_O_4_:Eu^2+^ particles are present to respond to the charge spread across the film surface. Thus, maintaining the SrAl_2_O_4_:Eu^2+^ content above a specified threshold enables reliable visualization of the charge distribution irrespective of the content level.

Based on the above results, when an SEL film is used as the active layer of a charge-detection sensor, increasing the SEL material content may improve the signal intensity. In contrast, the content ratio was found to have no significant effect on the relative change in luminescent area with applied voltage. This finding suggests that a high concentration of SEL material may not necessarily be required when the primary objective is to visualize charge distribution. Overall, these findings suggest that optimizing the SEL material concentration could be a key factor in designing charge-distribution sensing systems that balance signal intensity and visualization performance in the future.

## 4. Conclusions

In this study, we fabricated SEL films containing SrAl_2_O_4_:Eu^2+^ and evaluated their luminescence characteristics. High-voltage tests using needle electrodes revealed that the maximum SEL intensity occurred directly beneath the electrode, and the luminescence area expanded radially outward from that point. Considering the relationship between applied voltage and SEL response, SEL intensity was found to be proportional to the square of the applied voltage, whereas the luminescent area increased continuously and uniformly. These results suggest that SEL intensity may quantitatively reflect the amount of charge injected from the needle electrode. Furthermore, the luminescent area may correspond to the charge distribution on the film.

We also examined how SrAl_2_O_4_:Eu^2+^ content affects the luminescence characteristics of the SEL film. The findings of this experiment indicate two primary possibilities: First, increasing the SrAl_2_O_4_:Eu^2+^ content is possible to increase the signal intensity. Second, a high SrAl_2_O_4_:Eu^2+^ content is not necessarily required when the primary objective is visualization of the charge distribution.

These results suggest that optimizing SEL films incorporating SrAl_2_O_4_:Eu^2+^ may contribute to the development of a technique for sensitive, real-time visualization of charge distributions. Furthermore, the findings provide fundamental insights for the future development of self-contained charge-detection sensors and may contribute to addressing electrostatic issues in industrial environments.

## Data Availability

The original contributions presented in this study are included in the article/Supplementary Material. Further inquiries can be directed to the corresponding author.

## Supplementary Materials

The following supporting information can be downloaded at: https://www.mdpi.com/article/10.3390/ma19132709/s1. Figure S1: Integrated luminescence images acquired upon application of pulse voltages of (a) 4 kV, (b) 6 kV, (c) 7 kV, (d) 8 kV, and (e) 9 kV to the needle electrode in a sample containing 50% SrAl_2_O_4_:Eu^2+^. Corresponding vertical and horizontal SEL intensity profiles for (f) 4 kV, (g) 6 kV, (h) 7 kV, (i) 8 kV, and (j) 9 kV applied to the needle electrode, respectively.
