# Supplementary material for: Static Electricity-Induced Luminescence Materials for Charge Sensing
Source: Materials (Basel). 2026 Jun 24;19(13):2709. doi: 10.3390/ma19132709 (PMC13362577; doi:10.3390/ma19132709)
Supplement: Supplementary file 1 [file materials-19-02709-s001.zip › materials-4332975-supplementary.pdf]

Supplementary Materials for

Static Electricity-Induced Luminescence Materials for Charge Sensing

Tomoya Sato [1,*], Taiga Eguchi [1], Nanami Ishizu [1,2], Yuki Fujio [1] and Kazuya Kikunaga [1]

[1] Sensing Technology Research Institute, National Institute of Advanced Industrial Science and Technology, 807-1 Shuku-Machi, Tosu 841-0052, Japan; eguchi-0616@aist.go.jp (T.E.); yuki-fujio@aist.go.jp (Y.F.); k-kikunaga@aist.go.jp (K.K.)
[2] Faculty of Science and Engineering, Saga University, 1 Honjo, Saga 840-8502, Japan
* Correspondence: tmy-sato@aist.go.jp

Supplementary figures

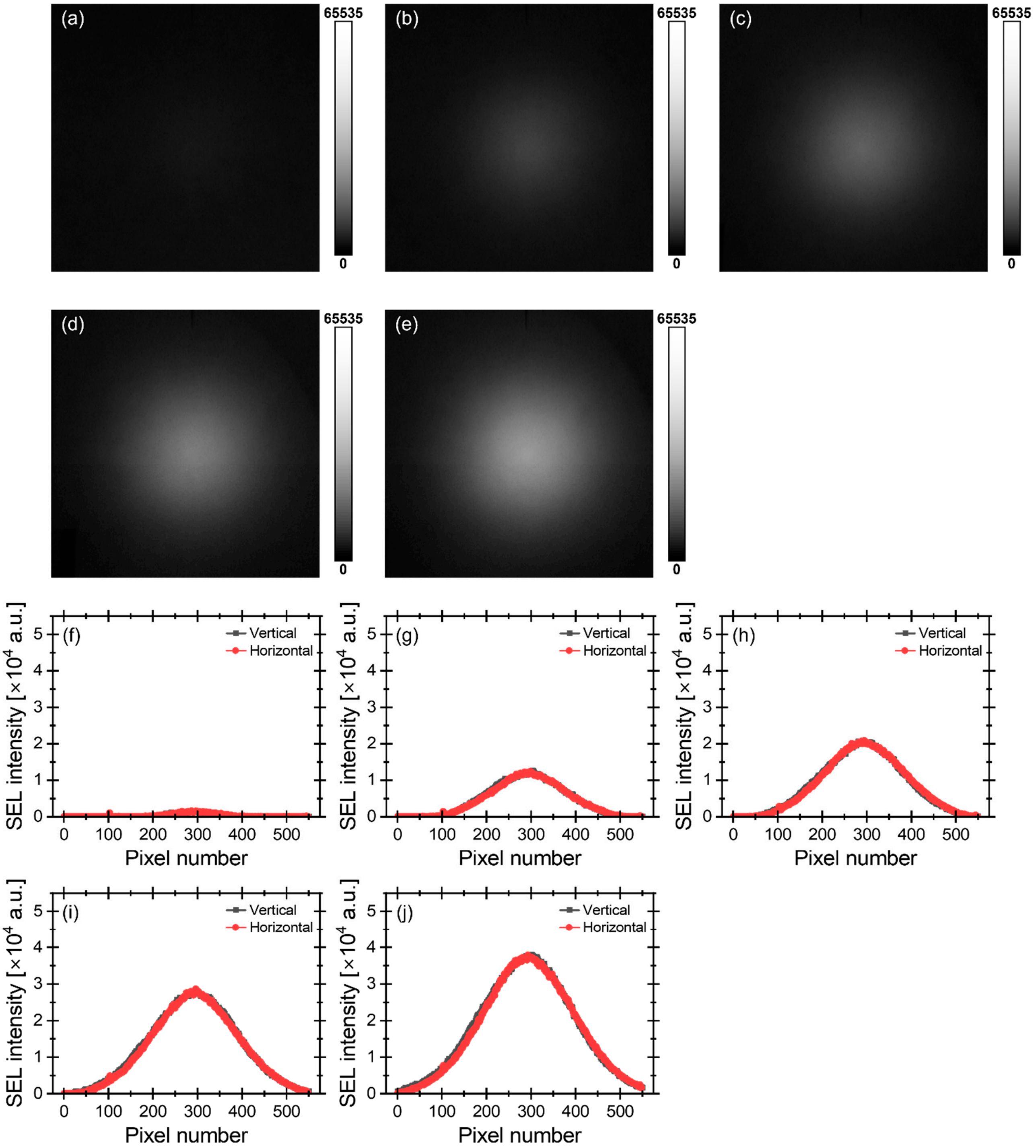

Figure S1. Integrated luminescence images acquired upon application of pulse voltages of (a) 4 kV, (b) 6 kV, (c) 7 kV, (d) 8 kV, and (e) 9 kV to the needle electrode in a sample containing 50% $SrAl_2O_4:Eu^{2+}$. Corresponding vertical and horizontal SEL intensity profiles for (f) 4 kV, (g) 6 kV, (h) 7 kV, (i) 8 kV, and (j) 9 kV applied to the needle electrode, respectively.
